# Self-Reported Fears by Hospice Patients at the End of Life

**DOI:** 10.1093/geroni/igaa057.795

**Published:** 2020-12-16

**Authors:** Jeannette Kates

**Affiliations:** Thomas Jefferson University, Philadelphia, Pennsylvania, United States

## Abstract

Fear is a common emotion that involves the intense anticipation of threat to a person. At end of life, this fear is often conceptualized as existential distress, which suggests a connection to spirituality. Processing impending death is essential to end-of-life closure and acceptance. Existing evidence suggests that spirituality is associated with greater coping, better psychosocial well-being, and dignified dying; however, the relationship between fear and spirituality at end of life, as well as the specific fears experienced, are not known. The purpose of this study was to explore the relationship between fear and spirituality in patients upon hospice admission. In this retrospective study, admission records from 154 hospice patients were reviewed. Hospice admission data from the psychosocial and spiritual assessments were analyzed using descriptive statistics, inferential statistics, and logistic regression. The average patient age was 81 years of age. A slight majority (51.3%) of patients admitted to fears upon hospice admission. Patients reported a range of one to six fears, with the most common fear being “pain and/or suffering.” Forty-seven percent of the patients identified as being “spiritually active.” Correlation analysis revealed no statistically significant relationship between fear and spiritually. Logistic regression analysis revealed some significant relationships between age and certain fears. Fear is a common symptom at the end of life, and appropriate emotional and psychological support should be provided to mitigate the fears. This study suggests that fears may be different for older adults.

